# Fecal Microbiota Transplantation for Chronic Pouchitis: A Systematic Review and Meta-Analysis

**DOI:** 10.3390/microorganisms12122430

**Published:** 2024-11-26

**Authors:** Magnus Chun, Kyaw Min Tun, Tahne Vongsavath, Renuka Verma, Kavita Batra, David Limsui, Erin Jenkins

**Affiliations:** 1 Department of Internal Medicine, Kirk Kerkorian School of Medicine at UNLV, University of Nevada, Las Vegas, NV 89106, USA; tahne.vongsavath@unlv.edu (T.V.); renuka.verma@unlv.edu (R.V.); 2 Division of Gastroenterology and Hepatology, Department of Internal Medicine, School of Medicine, Creighton University, Omaha, NE 68178, USA; danielkyawmintun@gmail.com (K.M.T.); erinjenkins@creighton.edu (E.J.); 3 Department of Medical Education, Kirk Kerkorian School of Medicine at UNLV, University of Nevada, Las Vegas, NV 89106, USA; 4 Office of Research, Kirk Kerkorian School of Medicine at UNLV, University of Nevada, Las Vegas, NV 89106, USA; 5 Division of Gastroenterology and Hepatology, Department of Medicine, School of Medicine, Stanford University, Palo Alto, CA 94305, USA; dlimsui@stanford.edu

**Keywords:** fecal microbiota transplantation, FMT, ulcerative colitis, inflammatory bowel disease, IBD, pouchitis

## Abstract

Pouchitis is a common complication after ileal-pouch anal anastomosis in patients with medically refractory ulcerative colitis. There has been a lack of high-level evidence focusing on the safety and efficacy outcomes of fecal microbiota transplantation (FMT). We aim to evaluate outcomes and complications of fecal microbiota transplantation (FMT) for chronic pouchitis. Databases were systematically searched to retrieve English-only, original studies, published from inception to 31 March 2024, investigating chronic pouchitis only. Primary outcomes included overall remission, clinical response, remission, relapse, and complications. Seven studies with 94 patients were included. The pooled overall remission rate was 15% (95% CI: 0–29%, *p* < 0.001), the clinical response rate was 33% (95% CI: 19–46%, *p* = 0.14), the clinical remission rate was 14% (95% CI: 19–46%, *p* < 0.001), and the clinical relapse rate was 36% (95% CI: 16–55%, *p* = 0.11). The pooled proportion of patients with mild adverse events after FMT treatment was 39% (95% CI: 6–71%, *p* < 0.001). No severe adverse events or deaths were reported. Although FMT is an effective treatment for chronic pouchitis, there is still a high rate of mild adverse events. High-level evidence for FMT is still sparse, limiting recommendations for clinical use.

## 1. Introduction

Inflammatory bowel diseases (IBDs) such as ulcerative colitis (UC) and Crohn’s disease (CD) are chronic autoimmune inflammatory conditions of the gastrointestinal tract with dysregulated immune responses to an altered gut microbiome. Despite recent advances in medical therapies for IBDs, many patients may eventually require surgical interventions. Ileal-pouch anal anastomosis (IPAA) has been one of the most common restorative surgical interventions for medically refractory UC [1]. A common complication following this procedure is pouchitis, which can occur in up to 60% of UC patients [2]. Symptoms of pouchitis include increased stool urgency and frequency as well as abdominal and pelvic pain.

The current guidelines for the treatment of pouchitis is continuous antibiotic treatment for several weeks, usually with ciprofloxacin and/or metronidazole. However, ten to fifteen percent of these patients develop chronic pouchitis and often require long-term antibiotics, advanced immunosuppressive therapies, or surgical revision. Therapies for chronic pouchitis can be difficult to manage for most patients [3,4]. While the pathogenesis of pouchitis is poorly understood, there have been studies showing involvement of the fecal microbiota [5,6]. The diet that patients intake is closely related to the microbial metabolites and intestinal flora compositions. However, patients with IBD were found to have decreased floral diversity and an increased dysregulation of microbiota in the intestines. Some studies have found an increase in *Enterococcus* and a decrease in *Akkermansia muciniphila, Firbicutes* and *Bacteroides*. *A. muciniphilia* is one of the most abundant normal human gut microbe, and studies have found that *A. muciniphilia* is decreased in UC patients [7]. Patients who have healthy low-carb, high-fat diets have less alteration of intestinal microbiota compared to patients with IBD [8,9]. The alteration of intestinal microbiota in IBD patients causes a decrease in intestinal defense function, allowing intestinal mucosal invasion and intestinal inflammatory responses. During inflammation, there is more oxygen delivered to the inflammatory site, causing the environment to be poisonous for obligate anaerobes and diminishes the gut mucus layer that originally contained healthy gut microbiota. This process known as “gut microbiota dysbiosis” is the etiology of the symptoms of UC including bloody diarrhea, abdominal pain, urge to defecate, and changes in bowel habits. Theoretically, replacing the altered gut microbiota with healthy gut microbiota can improve symptoms of UC [9]. As a result, there has been an increased attention on potential therapies that modify the fecal microbiome in patients with chronic pouchitis [10].

Fecal microbiota transplantation (FMT) is a fecal suspension taken from a healthy individual to be infused into the gastrointestinal tract of a chronic pouchitis patient. Given the potential to restore normal intestinal microbiota, FMT has emerged as a novel potential therapy for chronic pouchitis as well as other gastrointestinal disorders. FMT has been known to replace inflammatory pathogenic bacterium with those who upregulate fatty chain acids, vitamins, and amino acids, decreasing gut barrier permeability and immune system regulation [11]. By allowing the reintroduction of normal flora, FMT assists to reverse the decreased alpha-diversity related to pouchitis. FMT has found utility in treating disease related to the gut microbiota, such as recurrent *Clostridioides difficile* infection (rCDI), and it is more recently being studied for use in antibiotic-associated diarrhea [12]. The possibility of FMT improving quality of life and reducing risks of antibiotic resistance and side effects associated with current long-term therapies for chronic pouchitis is an attractive option for patients. Recent studies have shown FMT to be a safe and effective treatment that can significantly improve symptoms in chronic pouchitis [5,13,14,15,16,17,18]. Our systematic review and meta-analysis provides an assessment of the current literature on FMT therapy for chronic pouchitis with an emphasis on clinical outcomes and adverse events.

## 2. Materials and Methods

### 2.1. Search Strategy

This paper was systematically searched according to Preferred Reporting Items for Systematic Reviews and Meta-Analysis (PRISMA) [19]. The PubMed, Embase, Web of Science, CINAHL, Google Scholar, and Cochrane databases were systematically searched for all publications from inception to 31 March 2024. The following keywords were used in all databases: [“fecal microbiota transplant” AND “pouchitis” OR “chronic pouchitis”]. The full search strategy is available in Supplementary Materials. All English-language original studies were included in the search process that retrieved a total of 95 articles. These were exported and 32 duplicates were removed, leaving 63 studies to screen [20]. Further screening excluded 14 studies for non-FMT intervention and 6 studies for having a wrong study design. This ultimately left 7 studies to be included in the review. Further details of study selection provided by PRISMA is shown in Supplementary Materials. The template for the PRISMA checklist that was used for our study can be found in Supplementary Materials. The research protocol was created prior to conducting a formal search strategy. This study consisting of a systematic review and meta-analysis was registered on Prospero (ID 569557).

### 2.2. Study Selection

Relevant articles were independently reviewed by 2 authors (MC, RV). Any discrepancies in the article selection were arbitrated by a third author (KMT). We included studies that had outcomes of treatment of FMT in chronic pouchitis patients. We excluded non-FMT interventions for pouchitis, studies examining CD, literature reviews/letters/commentaries, case reports/case series, and non-human or cadaver studies. 

In our analysis, we aimed to assess the outcomes of FMT treatment in chronic pouchitis. The following questions were used to determine outcomes to be collected:

1. Can FMT help achieve overall remission (assessed with Pouchitis Disease Activity Index score (PDAI) < 7 at 4 weeks follow-up)?
2. Can FMT help achieve clinical response (assessed with the absolute difference of PDAI ≥ 3 between initial visit and at 4 weeks follow-up)?
3. Can FMT help achieve clinical remission (assessed with PDAI < 4 at 4 weeks follow-up and no need for antibiotics or subjective improvement in symptoms)?
4. Can FMT decrease risk of clinical relapse (assessed with the presence of recurrence of symptoms at 4 weeks follow-up)?
5. Are there any adverse events attributable to FMT noted up to 12 months after the treatment?

The composite score, PDAI, has been used to diagnose pouchitis and was included as an objective measurement in this study [21]. The PDAI score is calculated from three different six-point scales consisting of clinical symptoms, endoscopic findings, and histological changes. Clinical symptoms included increased stool frequency, abdominal cramps, rectal bleeding, and fever > 37.8 degrees Celsius. Endoscopic findings included edema, granularity, friability, loss of vascular pattern, mucous exudates, and ulcerations. Histological changes seen on biopsy taken during endoscopy included polymorphonuclear infiltration and ulceration per low power field. A cut-off value of 7 differentiates between “active pouchitis” and “no pouchitis” (Supplementary Materials). 

### 2.3. Data Extraction

Two authors (MC, TV) independently screened and extracted data in two steps: (1) titles and abstracts, and (2) full-text articles. Data from each relevant study were extracted into a standardized form which included (1) article author, (2) year of publication, (3) study design, (4) quality of study, (5) sample size, (6) demographics, (7) FMT treatment protocol with route, dosage, and duration, (8) primary outcomes (PDAI at initial visit and PDAI at follow-up visits), (9) length of follow-up, (10) adverse events, and (11) secondary outcomes (inflammatory markers, stool frequency, rectal bleeding at initial visit and 4 weeks follow-up). Adverse events were classified by the ASGE severity grading scale [22]. The National Institute of Health Quality Assessment Tool was used to determine study quality with ratings categorized as “Excellent”, “Good”, and “Poor” (Supplementary Materials) [23].

### 2.4. Outcomes and Effect Size

The primary outcomes of this meta-analysis was the pooled proportion of overall remission, clinical response, clinical remission, clinical relapse, and adverse events. The secondary outcomes included overall remission and clinical response based on the country of study and donor source.

### 2.5. Statistical Analysis

Individual estimates of each study were pooled to compute the summary estimates of the clinical outcomes using the restricted maximum likelihood estimation method. A random model accounting for both within- and between-study variance was fit for generating summary estimates [24]. The strength of evidence of heterogeneity across studies was determined by Cochran’s Q and I2 statistics [24]. The Cochran’s Q test follows a Chi-square (χ^2^) distribution. The degrees of freedom (df) for this distribution are equal to the number of studies (k) minus one (k − 1). If the Q statistic is significantly larger than expected under the Chi-square distribution, it suggests that the variability among study results cannot be explained by chance alone, indicating the presence of heterogeneity. The values of under 30%, 30–60%, 61–75%, and over 75% were categorized as low, moderate, substantial, and considerable heterogeneity, respectively [25]. Subgroup analyses of the clinical outcomes by etiology were also performed. Sensitivity analysis was conducted to determine the validity of the estimated summary effect size. For the sensitivity analysis, a “leave-one-out analysis” was conducted to investigate the impact of removal of study (one by one) on the estimates and LFK asymmetry. In addition, the Luis Furuya–Kanamori (LFK) index was used as a quantitative method to assess the asymmetry of the study effects or publication bias, as it has been noted in the literature that the LFK index has higher sensitivity than the Egger regression statistics, particularly in the meta-analysis with small numbers of studies [23,26]. All meta-analyses were performed using MetaXL software (v. 5.3; EpiGear International, Sunrise Beach, Queensland, Australia) and SPSS (version 28). The 95% Clopper–Pearson exact confidence intervals were calculated using the R package version 4.4.2. 

## 3. Results

### 3.1. Search Results and Demographics

A total of seven studies with 94 patients receiving FMT for chronic pouchitis were included in our meta-analysis [5,13,14,15,16,17,18]. All seven studies were prospective cohort studies published between 2015 and 2021: three from the USA, one from Germany, one from the United Kingdom, one from Denmark, and one from Finland. The sample size between the studies ranged from 6 to 26 years old. There were more female patients than males with a total of 33 males and 45 females. The gender of 16 patients was not documented. The study follow-up time ranged from 1 to 37 months (mean: 29.6 months) The demographics and quality assessment of the included studies are described in Table 1. Based on the National Institute of Health Quality Assessment Tool, all of the included studies were rated as “good” quality (Table 1). 

### 3.2. Study Characteristics

The FMT route of administration varied between studies with three studies using pouchoscopy, one using nasogastric tubes, one using capsules, one using enemas, and one using a combination of pouchoscopy and trans-anal catheter. Probiotics were used in one of the seven studies included [13]. However, antibiotics prior to FMT were used in three of the studies [5,15,18]. Antibiotics were administered at various times during the FMT treatment including prior to, during, and afterwards. The studies did not clarify when antibiotics were administered. Some studies had administered antibiotics before FMT and some were after. Studies varied in the frequency of FMT given during the study period. Three studies administered FMT only once [13,16,18]. Two studies administered FMT two times with a 4-week interval between the administrations [15,17]. Steube et al. had 5 consecutive days of FMT given, while Kousgaard et al. had up to 14 consecutive days of FMT given [14,15]. The FMT treatment protocol for the included studies is detailed in Table 2. The process of selecting FMT donors varied and is detailed in Table 2. The screening and distribution process in order to obtain a stable and reproducible FMT solution varied across studies, but some of the studies used non-profit organizations, such as OpenBiome. El Nachef et al. and Selvig et al. used OpenBiome to obtain stool samples [5,16]. OpenBiome distributes fecal microbiota preparation samples under the Investigational New Drug application. The results and other clinical characteristics are included in Supplementary Materials. Mild adverse events of the included studies are noted in Supplementary Materials. No severe adverse events or deaths were reported. 

### 3.3. Meta-Analysis of Primary Outcomes

**Figure 1:**
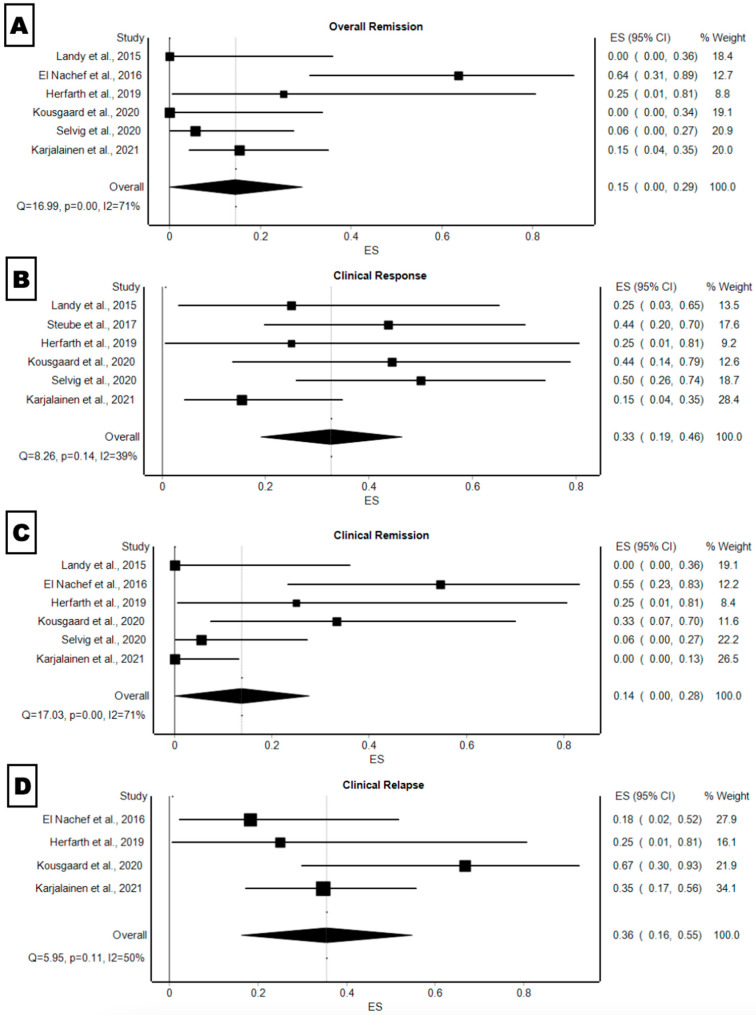
FMT treatment forest plots of (**A**) pooled proportion of overall remission, (**B**) pooled proportion of clinical response, (**C**) pooled proportion of clinical remission, and (**D**) pooled proportion of clinical relapse [5,13,14,15,16,17,18].

Patients with pouchitis who achieved a PDAI < 7 at their 4 weeks follow-up after receiving FMT were classified to be in overall remission [24]. The pooled proportion of patients with overall remission was 15% (95% CI: 0–29%, *p* < 0.001, I2 = 71%) (Figure 1A). Patients with pouchitis who achieved an absolute difference of PDAI ≥ 3 between the initial visit and at 4 weeks follow-up after receiving FMT were classified as having a clinical response. The pooled proportion of patients with clinical response was 33% (95% CI: 19–46%, *p* = 0.14, I2 = 39%) (Figure 1B). Patients with pouchitis who achieved a PDAI < 4 at 4 weeks follow-up and no longer needed antibiotics or had a subjective improvement in symptoms after receiving FMT were classified as having clinical remission [15].The pooled proportion of patients with clinical remission was 14% (95% CI: 19–46%, *p* < 0.001, I2 = 71%) (Figure 1C). Patients with pouchitis who had a recurrence of symptoms at 4 weeks follow-up after receiving FMT were classified as having clinical relapse. The pooled proportion of patients with clinical relapse (recurrence of symptoms at week 4 follow-up appointment after treatment began) was 36% (95% CI: 16–55%, *p* = 0.11, I2 = 50%) (Figure 1D). The frequency of FMT administration had no correlation with outcomes.

**Figure 2:**
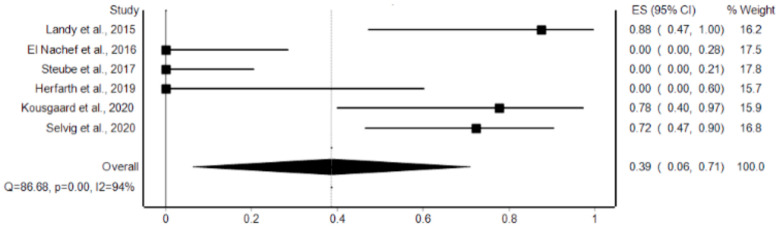
Forest plot of pooled proportion of complications/adverse events for FMT treatment at 4 week follow-up [5,13,14,15,16,18].

In patients receiving FMT, the pooled proportion of patients with mild adverse events as classified by the ASGE severity grading scale was 39% (95% CI: 6–71%, *p* < 0.001, I2 = 94%) with abdominal pain and nausea/vomiting being the most prevalent (Figure 2). All mild adverse events reported lasted less than 14 days after FMT treatment. No severe adverse events or deaths were reported. No adverse events led to cessation of therapy or hospital admission. Out of the total 94 patients, 16 patients complained of abdominal pain, 9 complained of nausea/vomiting, 5 patients reported bloating, 3 patients reported fever, and 3 patients reported fatigue (Supplementary Materials). However, three studies reported no adverse events experienced by their patients, while Selvig et al. reported more than 90% of patients experiencing adverse events [5,14,16,18].

### 3.4. Subgroup Analysis

Subgroup analysis was conducted by the country in which the study took place and donor source (single versus multiple). The only primary outcomes that were analyzed were overall remission and clinical response.

**Figure 3:**
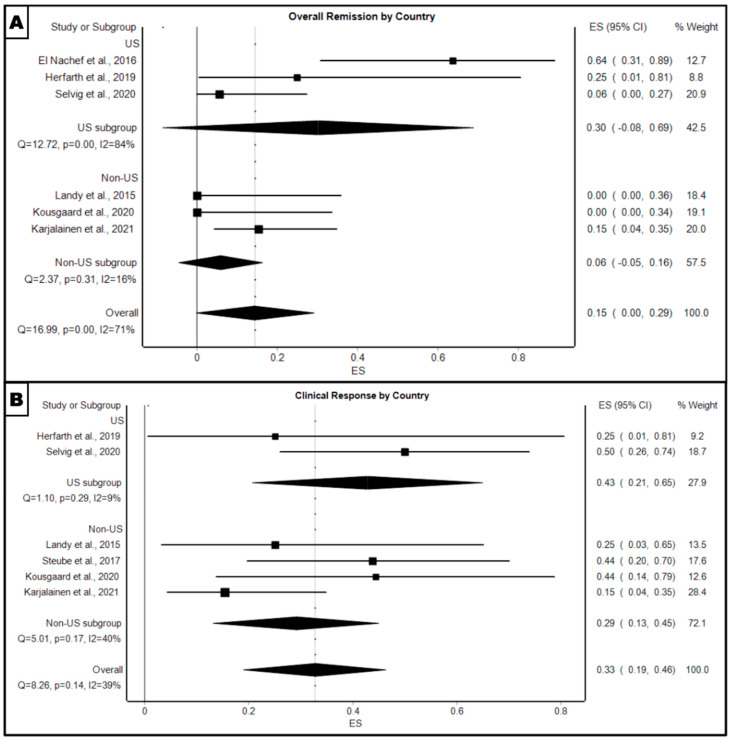
Forest plot of pooled proportion of (**A**) overall remission for FMT treatment at 4 week follow-up, moderated by country in which the study took place, and (**B**) clinical response for FMT treatment at 4 week follow-up, moderated by country in which the study took place [5,13,14,15,16,17,18].

The pooled proportion of patients in the US subgroup had a higher overall remission rate of 30% (95% CI: −8–69%, *p* < 0.001) at 4 weeks follow-up compared to the non-US subgroup (6%, 95% CI: −5–16%, *p* = 0.31) (Figure 3A). Additionally, the pooled proportion of patients in the US subgroup had a higher clinical response rate of 43% (95% CI: 21–65%, *p* = 0.029) at 4 weeks follow-up compared to the non-US subgroup (29%, 95% CI: 13–45%, *p* = 0.17) (Figure 3B).

**Figure 4:**
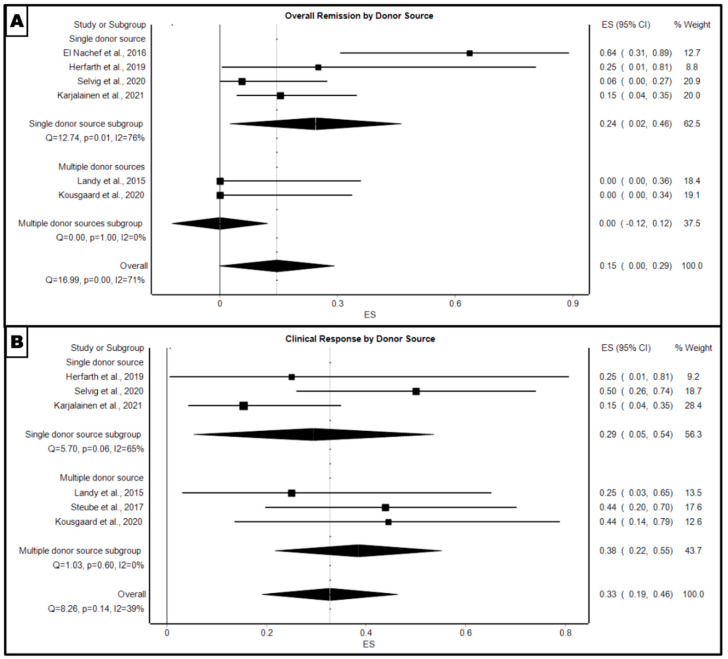
Forest plot of pooled proportion of (**A**) overall remission for FMT treatment at 4 week follow-up, moderated by donor source (single versus multiple donor subgroups) and (**B**) clinical response for FMT treatment at 4 week follow-up moderated by donor source (single versus multiple donor subgroups) [5,13,14,15,16,17,18].

FMT with a single donor source had a higher overall remission rate (24%, 95% CI: 2–46%, *p* = 0.01) compared to multiple donor sources (0%, 95% CI: −12–12%, *p* = 1) (Figure 4A). However, FMT with multiple donor sources had a higher overall clinical response (38%, 95% CI: 22–55%, *p* = 0.6) compared to single donor sources (29%, 95% CI: 5–54%, *p* = 0.06) (Figure 4B).

### 3.5. Validation of Meta-Analysis Results

The meta-regression analysis reported no significant modifier covariates that would influence the results reported in our included studies. Regarding sensitivity analysis, no significant differences were found (Supplementary Materials). Egger’s regression tests showed no evidence of publication bias (Supplementary Materials). 

## 4. Discussion

In our meta-analysis which included a total of seven studies with ninety-four patients receiving FMT for chronic pouchitis, we determined the clinical response rate of FMT therapy to be 33% at 4 weeks follow-up and 39% of patients experienced mild adverse effects that resolved within 14 days of FMT therapy. The included studies found FMT therapy to be well tolerated overall with no reported severe adverse events leading to hospitalization or death. 

FMT has the potential to be therapeutic in patients with chronic pouchitis, but having an established standardized protocol would be advantageous. The efficacy of FMT may vary depending on the routes of administration such as colonoscopy, sigmoidoscopy, esophagogastroduodenoscopy, enteroscopy, percutaneous endoscopic cecostomy, jejunostomy, nasogastric tube, transendoscopic enteral tube, and enema [11]. Our meta-analysis included a variety of FMT routes for chronic pouchitis. Furthermore, no set protocol exists for treatment of pouchitis with FMT; thus, different means of delivery, amount of stool, and number of sessions of FMT transplant were utilized in each study. Additional research is required to help establish a significant correlation between route of FMT and complications/outcomes.

Based on our meta-analysis, the frequency of FMT sessions did not significantly affect outcomes of remission, response, or complications. However, in Kousgaard et al., the continuous daily FMT administration seemed to improve the clinical remission by the 7th day of administration [15]. This could indicate that FMT for chronic pouchitis requires several FMT treatments to achieve clinical remission, whereas usually one dose of FMT is sufficient to treat *Clostridioides difficile* infection. 

The FMT donors varied between single (4/7) and multiple (3/7) in the studies included. Multiple donors were used in the studies conducted by Landy et al. and Kousgaard et al. and did not report any cases of overall remission [13,15]. In addition, Kousgaard et al. also reported the highest proportion of clinical relapse (6/9) as compared to studies with single donors [15]. Studies with single donors such as El-Nachef et al. reported better outcomes in overall remission (7/7) and clinical remission (6/11) [16]. In a previous study involving the use of FMT for rCDI patients, Cold et al. found that there were no differences in outcomes based on single vs. multi-donor treatments [27,28]. However, multiple mice and human studies found that some donor microbiota engraft better than others, indicating a possible need to individualize the microbiota screening of donors and patients prior to FMT to obtain the best match [15,18,29]. In regard to UC, Moayyedi et al. demonstrated improvement in clinical response with two donors with similar microbiota profiles compared to one donor. Single donors with similar microbiota with recipients had better clinical responses in UC than multi-donors who might have different microbiota than their recipients [30]. In prior studies, FMT responders demonstrated alpha diversity, defined as microbial richness, and beta diversity, defined as microbiota diversity, compared to non-responders, and sustained similarity of flora to donors during follow-up [12]. Studies suggest that patients with higher pre-treatment relative levels of butyrate-producing organisms such as *Blautia* and *Clostridiales* have an increased likelihood of response perhaps by creating a more viable environment for grafted bacterium. Some studies have shown a lack of *A. muciniphilia* in UC patients. Future studies can investigate whether FMT should have a diverse heterogenous gut microbiome or more of a homogenous microbiome, mainly including *A. muciniphilia*. It is also one of the most abundant normal human gut microbes, and if included in FMT’s microbiome, it would theoretically lead to FMT’s success in UC patients [7]. Patients with higher baseline levels of diversity have also been reported to be more likely to respond. This may be related to competition of the normal flora against the grafted flora or a reflection of depleted flora in patients with severe baseline inflammation [31]. However, there are limited published outcomes on single vs. multi-donor FMT for chronic pouchitis. While our study suggests some potential differences in remission and response, further studies are required to determine if there are any significant differences between single and multi-donor FMT treatment among patients with pouchitis and if there are other donor characteristics to be considered. 

Antibiotics and probiotics are routinely used in the treatment of pouchitis [32]. Similarly, amongst patients being treated with FMT, antibiotic pre-treatment in cases of IBD has been shown to improve the response and remission rates [33]. Among cases with pouchitis, in their study evaluating the impact of FMT, El-Nachef et al. found that the concurrent use of ciprofloxacin in a patient with the previous use of probiotics as well as rifaximin and ciprofloxacin led to an improvement in their GI symptoms as well as their PDAI score (9 to 5). However, in the cases that used probiotics, they did not report an improvement in gastrointestinal symptoms despite an improvement in their PDAI scores (9 to 6 and 6 to 2) [16]. All patients evaluated by Kousgaard et al. reported the use of either ciprofloxacin and/or metronidazole, amongst which four out of nine patients relapsed with a mean of 4.3 days, and another two relapsed after 15 and 52 days. However, it was unclear how long prior to FMT they received the antibiotics [15]. While our analysis may suggest that the prior or concurrent use of antibiotics may improve the outcomes of FMT in treating pouchitis, there is a paucity of randomized controlled trials comparing patients on antibiotics to those without [17,18,34]. The potential impact of antibiotic/probiotic use during FMT for chronic pouchitis should be further studied.

There are several strengths to our meta-analysis. We conducted a thorough literature search and rigorous evaluation of our included studies to validate the results of our meta-analysis. This meta-analysis provides an update that does not include case reports, and our outcomes were pooled based on uniform definitions of the clinical outcomes using PDAI. Prior literature reviews which included case reports and case series may have led to bias in outcomes. Adverse events were included to justify our conclusion of FMT treatment. Previous systematic reviews did not focus on safety outcomes. The limitations of our meta-analysis included a possible variation in definitions of clinical response and remission in our included studies. There may have been differences in subjective reporting of the improvement in symptoms of patients. However, we used the PDAI as an objective measurement of our outcomes, which is standardized among included studies. Each included study had different confounding variables within their protocol that could not be excluded in our meta-analysis. Additionally, it was not possible to conduct a two-arm meta-analysis because only one out of the seven included studies had data comparing the FMT group with a control group [16]. We were not able to compare adjunctive probiotic therapy because only one study included outcomes of FMT with adjunctive probiotic therapy. We noted moderate–high heterogeneity in our results.

In conclusion, although FMT may be considered an effective treatment for chronic pouchitis, there is a high rate of mild adverse events. High-level evidence for FMT is still sparse, which may limit recommendations for its use in clinical practice. More robust randomized controlled trials are needed to ultimately determine FMT’s efficacy in chronic pouchitis and its optimal delivery method, duration, and donor source.

## Figures and Tables

**Table 1:** Patient demographics and quality rating of included studies.

Study	Design	Country	Sample Size (N)	Age (y, Mean)	Males/Females (N)	NIH Study Quality
Steube et al., 2017 [14]	Prospective	Germany	16	NR	NR	Good
El Nachef et al., 2016 [16]	Prospective	USA	11	47 (27–77 range). [median]	3/8	Good
Landy et al., 2015 [13]	Prospective	UK	8	46 (24–63 range). [median]	3/5	Good
Herfarth et al., 2019 [18]	Prospective	USA	6 *	37.3 (22–60 range). [median]	4/2	Good
Kousgaard et al., 2020 [15]	Prospective	Denmark	9	51.5 (13.9 SD)	3/6	Good
Selvig et al., 2020 [5]	Prospective	USA	18	45 (34.3–56.3 range). [median]	6/12	Good
Karjalainen et al., 2021 [17]	Prospective	Finland	26	42.7 (10.2 SD)	14/12	Good

NR—not recorded. *—4 patients received FMT and 2 received placebo.

**Table 2:** FMT treatment protocol.

Study	Donor Selection Process	Patient Bacterial Composition	FMT Treatment Duration	FMT Treatment Route	FMT Donor Source	FMT Treatment Dosage	Duration of Follow-Up (Weeks)
Steube et al., 2017 [14]	Stool sample obtained from unknown. Donor was unrelated to recipient.	NR	5 consecutive days of FMT	Two possible routes: encapsulated or endoscopic jejunal application	Multiple	NR	NR
El Nachef et al., 2016 [16]	Stool sample obtained from OpenBiome. Donor was unrelated to recipient.	Pre-FMT: Enriched with *Clostridiaceae, Erysipelotrichaceae,* and *Enterobacteriace* Post-FMT: Enriched with *Streptococcus*, *Bacteroides*, and *Enterobacteriace*	Once-only dose of FMT	Pouchoscopy	Single	NR	NR
Landy et al., 2015 [13]	Stool sample obtained from unknown. Donor was unrelated to recipient.	Pre-FMT: Enriched with *Clostridiaceae* and *Enterobacteriace* Post-FMT: Enriched with *Sutterella stercoricanis*, *Dorea longicatena*, and *Faecalibacterium*	Once-only dose of FMT-saline solution	Nasogastric infusion	Multiple	30 g of fecal material in 50 mL normal saline	4
Herfarth et al., 2019 [18]	Stool sample obtained from unknown. Donor was unrelated to recipient.	NR	Once-only dose of FMT	Endoscopy initially and if clinical relapse would be followed by daily oral encapsulated FMT for 2 weeks	Single	Initial fecal material dose of 24 g followed by daily 4.2 g fecal material capsules	20
Kousgaard et al., 2020 [15]	Stool sample obtained from volunteers at study hospital. Donor was unrelated to recipient.	Pre-FMT: Not recorded Post-FMT: Enriched with *Bacteroides*	14 consecutive days of suspended FMT	Enema	Multiple	100 mL suspended fecal material	24
Selvig et al., 2020 [5]	Stool sample obtained from OpenBiome. Donor was unrelated to recipient.	Pre-FMT: Enriched with *Lacnospirarceae, Erysipelotrichaceae, Lachnoclostridium, Flavonifractor* and *Enterobacteriace* Post-FMT: Not recorded	FMT infusion initially infused into proximal pouch followed by second administration 4 weeks later	Pouchoscopy	Single	Initial: 25 g fecal material in 250 mL normal saline 4 weeks later: 50 g fecal material in 500 mL normal saline	48
Karjalainen et al., 2021 [17]	Stool sample obtained from one volunteer at study hospital. Donor was unrelated to recipient.	NR	Initial dose of FMT and second dose after 4 weeks	Pouchoscopy initially and second dose via trans-anal catheter	Single	Suspended fecal material in 100 mL normal saline	52

NR—not recorded.

## Data Availability

The original contributions presented in the study are included in the article/Supplementary Materials, further inquiries can be directed to the corresponding authors.

## Supplementary Materials

The following supporting information can be downloaded at: https://www.mdpi.com/article/10.3390/microorganisms12122430/s1, Table S1. Database search strategies for FMT therapy for chronic pouchitis. Table S2. PRISMA checklist. Table S3. The PDAI score is calculated from three different six-point scales comprising of clinical symptoms, endoscopic findings, and histological changes. Table S4. Table for Quality Assessment Tool for Observational Cohort Studies based on National Institute of Health Quality Assessment Tool. Table S5. Clinical characteristics of included studies. Table S6. Complications/Adverse events of included studies. Table S7. Table of sensitivity analysis. Figure S1. PRISMA flow diagram for study selection. Figure S2. Publication bias assessment. Egger’s regression test was used for the 7 included studies.
