# Supplementary material for: Fecal Microbiota Transplantation for Chronic Pouchitis: A Systematic Review and Meta-Analysis
Source: Microorganisms. 2024 Nov 26;12(12):2430. doi: 10.3390/microorganisms12122430 (PMC11676736; doi:10.3390/microorganisms12122430)

Supplementary Materials

Table S1. Database search strategies for FMT therapy for chronic pouchitis

Database: PubMed, Embase, Web of Science, CINAHL, Google Scholar, and Cochrane, In-Process and Other Non-Indexed Citations and Daily <1950 to 31 March 2024><br><br>Search Strategy:	
1	(Fecal Microbiota Transplant or FMT) AND (pouchitis or chronic pouchitis)

Figure S1. PRISMA flow diagram for study selection.

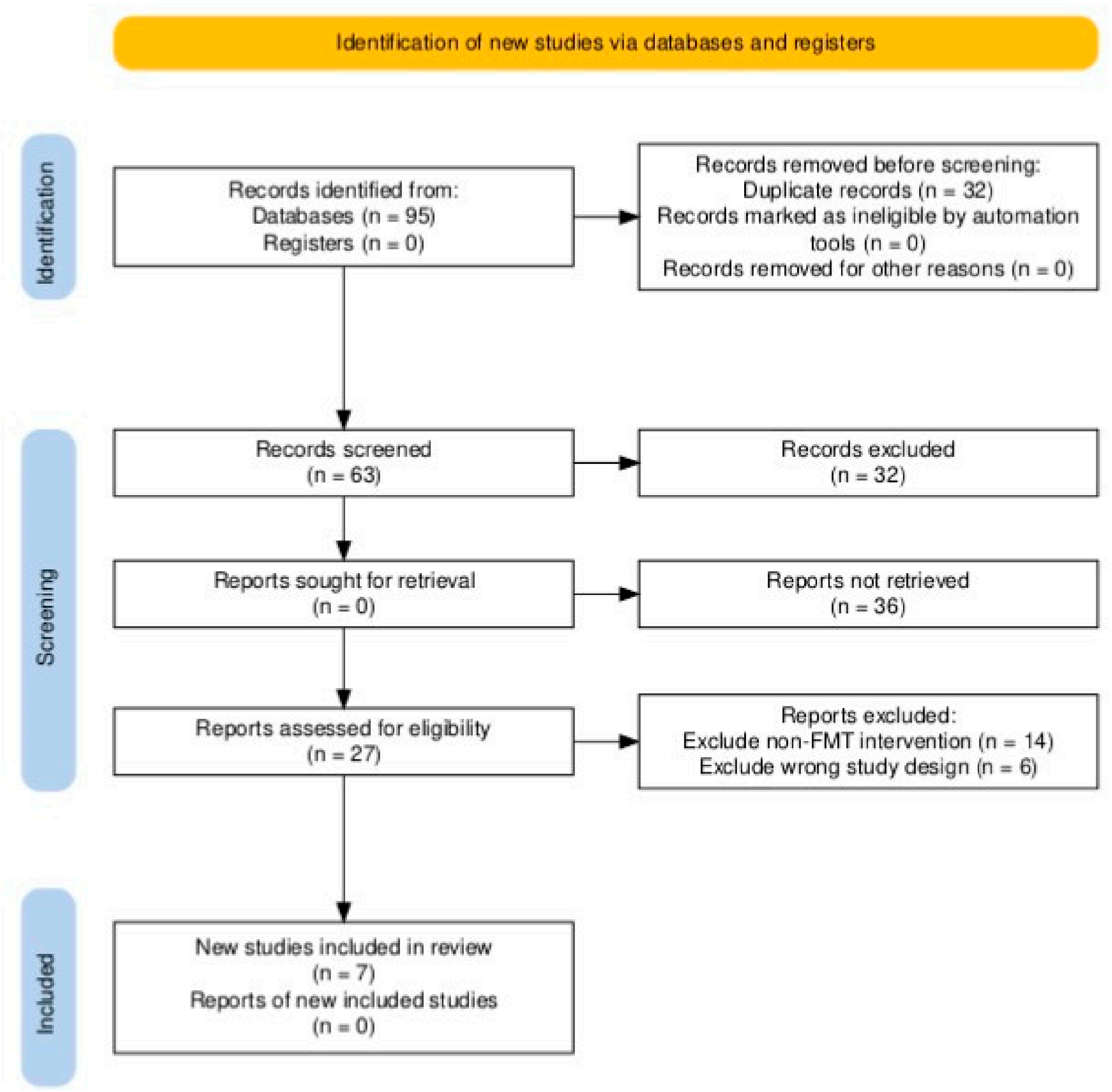

Table S2. PRISMA checklist

Section and Topic	Item #	Checklist item	Location where item is reported
**TITLE**			
Title	1	Identify the report as a systematic review.	
**ABSTRACT**			
Abstract	2	See the PRISMA 2020 for Abstracts checklist.	
**INTRODUCTION**			
Rationale	3	Describe the rationale for the review in the context of existing knowledge.	
Objectives	4	Provide an explicit statement of the objective(s) or question(s) the review addresses.	
**METHODS**			
Eligibility criteria	5	Specify the inclusion and exclusion criteria for the review and how studies were grouped for the syntheses.	
Information sources	6	Specify all databases, registers, websites, organisations, reference lists and other sources searched or consulted to identify studies. Specify the date when each source was last searched or consulted.	
Search strategy	7	Present the full search strategies for all databases, registers and websites, including any filters and limits used.	
Selection process	8	Specify the methods used to decide whether a study met the inclusion criteria of the review, including how many reviewers screened each record and each report retrieved, whether they worked independently, and if applicable, details of automation tools used in the process.	
Data collection process	9	Specify the methods used to collect data from reports, including how many reviewers collected data from each report, whether they worked independently, any processes for obtaining or confirming data from study investigators, and if applicable, details of automation tools used in the process.	
Data items	10a	List and define all outcomes for which data were sought. Specify whether all results that were compatible with each outcome domain in each study were sought (e.g. for all measures, time points, analyses), and if not, the methods used to decide which results to collect.	
	10b	List and define all other variables for which data were sought (e.g. participant and intervention characteristics, funding sources). Describe any assumptions made about any missing or unclear information.	
Study risk of bias assessment	11	Specify the methods used to assess risk of bias in the included studies, including details of the tool(s) used, how many reviewers assessed each study and whether they worked independently, and if applicable, details of automation tools used in the process.	
Effect measures	12	Specify for each outcome the effect measure(s) (e.g. risk ratio, mean difference) used in the synthesis or presentation of results.	
Synthesis methods	13a	Describe the processes used to decide which studies were eligible for each synthesis (e.g. tabulating the study intervention characteristics and comparing against the planned groups for each synthesis (item #5)).	

	13b	*Describe any methods required to prepare the data for presentation or synthesis, such as handling of missing summary statistics, or data conversions.*	
	13c	*Describe any methods used to tabulate or visually display results of individual studies and syntheses.*	
	13d	*Describe any methods used to synthesize results and provide a rationale for the choice(s). If meta-analysis was performed, describe the model(s), method(s) to identify the presence and extent of statistical heterogeneity, and software package(s) used.*	
	13e	*Describe any methods used to explore possible causes of heterogeneity among study results (e.g. subgroup analysis, meta-regression).*	
	13f	*Describe any sensitivity analyses conducted to assess robustness of the synthesized results.*	
Reporting bias assessment	14	*Describe any methods used to assess risk of bias due to missing results in a synthesis (arising from reporting biases).*	
Certainty assessment	15	*Describe any methods used to assess certainty (or confidence) in the body of evidence for an outcome.*	
RESULTS			
Study selection	16a	*Describe the results of the search and selection process, from the number of records identified in the search to the number of studies included in the review, ideally using a flow diagram.*	
	16b	*Cite studies that might appear to meet the inclusion criteria, but which were excluded, and explain why they were excluded.*	
Study characteristics	17	*Cite each included study and present its characteristics.*	
Risk of bias in studies	18	*Present assessments of risk of bias for each included study.*	
Results of individual studies	19	*For all outcomes, present, for each study: (a) summary statistics for each group (where appropriate) and (b) an effect estimate and its precision (e.g. confidence/credible interval), ideally using structured tables or plots.*	
Results of syntheses	20a	*For each synthesis, briefly summarise the characteristics and risk of bias among contributing studies.*	
	20b	*Present results of all statistical syntheses conducted. If meta-analysis was done, present for each the summary estimate and its precision (e.g. confidence/credible interval) and measures of statistical heterogeneity. If comparing groups, describe the direction of the effect.*	
	20c	*Present results of all investigations of possible causes of heterogeneity among study results.*	
	20d	*Present results of all sensitivity analyses conducted to assess the robustness of the synthesized results.*	
Reporting biases	21	*Present assessments of risk of bias due to missing results (arising from reporting biases) for each synthesis assessed.*	
Certainty of evidence	22	*Present assessments of certainty (or confidence) in the body of evidence for each outcome assessed.*	
DISCUSSION			
Discussion	23a	*Provide a general interpretation of the results in the context of other evidence.*	

	23b	*Discuss any limitations of the evidence included in the review.*	
	23c	*Discuss any limitations of the review processes used.*	
	23d	*Discuss implications of the results for practice, policy, and future research.*	
OTHER INFORMATION			
Registration and protocol	*24a*	*Provide registration information for the review, including register name and registration number, or state that the review was not registered.*	
	24b	*Indicate where the review protocol can be accessed, or state that a protocol was not prepared.*	
	24c	*Describe and explain any amendments to information provided at registration or in the protocol.*	
Support	*25*	*Describe sources of financial or non-financial support for the review, and the role of the funders or sponsors in the review.*	
Competing interests	*26*	*Declare any competing interests of review authors.*	
Availability of data, code and other materials	*27*	*Report which of the following are publicly available and where they can be found: template data collection forms; data extracted from included studies; data used for all analyses; analytic code; any other materials used in the review.*	

Table S3. The PDAI score is calculated from three different six-point scales comprising of clinical symptoms, endoscopic findings, and histological changes.

**Clinical findings**	**Range of scores**
Stool frequency	(0, 1, 2)
Fecal urgency/Abdominal cramps	(0, 1, 2)
Rectal bleeding	(0, 1)
Fever > 37.8 degrees Celsius	(0, 1)
**Endoscopic findings**	
Oedema	(0, 1)
Granularity	(0, 1)
Friability	(0, 1)
Loss of vascular pattern	(0, 1)
Mucous exudates	(0, 1)
Ulcerations	(0, 1)
**Histological findings**	
Polymorphonuclear infiltration	(0, 1, 2, 3)
Ulceration per low power field (mean)	(0, 1, 2, 3)

Table S4. Table for Quality Assessment Tool for Observational Cohort Studies based on National Institute of Health Quality Assessment Tool

**Criteria**	**Meets criteria? (1 point for each criteria)***
Was the research question or objective in this paper clearly stated?	
Was the research question or objective in this paper clearly stated?	
Was the participation rate of eligible persons at least 50%?	

Were all the subjects selected or recruited from the same or similar populations (including the same time period)? Were inclusion and exclusion criteria for being in the study prespecified and applied uniformly to all participants?	
Was a sample size justification, power description, or variance and effect estimates provided?	
For the analyses in this paper, were the exposure(s) of interest measured prior to the outcome(s) being measured?	
Was the timeframe sufficient so that one could reasonably expect to see an association between exposure and outcome if it existed?	
For exposures that can vary in amount or level, did the study examine different levels of the exposure as related to the outcome (e.g., categories of exposure, or exposure measured as continuous variable)?	
Were the exposure measures (independent variables) clearly defined, valid, reliable, and implemented consistently across all study participants?	
Was the exposure(s) assessed more than once over time?	
Were the outcome measures (dependent variables) clearly defined, valid, reliable, and implemented consistently across all study participants?	
Were the outcome assessors blinded to the exposure status of participants?	
Was loss to follow-up after baseline 20% or less?	
Were key potential confounding variables measured and adjusted statistically for their impact on the relationship between exposure(s) and outcome(s)?	

* - 1 to 5 points denotes "poor" study, 6 to 10 points denotes "good" study, and >10 points denotes "excellent" study

Table S5. Clinical characteristics of included studies

Study	Study	Clinical Remission (N)	Clinical Response (N)	Clinical Relapse (N)	Difference in Calprotectin levels after FMT treatment	Difference in CRP levels after FMT treatment	Difference in average stool frequency after FMT treatment	Difference in frequency of rectal bleeding after FMT treatment	Difference in frequency of stool urgency after FMT treatment
Steube et al., 2017[14]	NR	NR	7/16	NR	NR	NR	NR	NR	NR
El Nachef et al., 2016[16]	7/7	6/11	NR	2/7	NR	NR	-5.0	-1	-8
Landy et al., 2015[13]	0/8	0/8	2/8	NR	NR	NR	NR	NR	NR
Herfarth et al., 2019[18]	1/4	1/4	1/4	1/5	NR	NR	NR	NR	NR
Kousgaard et al., 2020[15]	0/9	3/9	4/9	6/9	-580	+5.6	-0.8	NR	NR
Selvig et al., 2020[5]	1/11	1/11	9/18	NR	-104	+3.7	-2.0	-2	0
Karjalainen et al., 2021[17]	4/13	0/13	4/13	9/13	-17	NR	0	0	0

Overall remission = PDAI < 7 at 4 weeks follow-up, Clinical remission = PDAI < 4 at 4 weeks follow-up and no need for antibiotics or subjective improvement in symptoms, Clinical response = absolute difference of PDAI ≥ 3 between initial visit and at 4 weeks follow-up, Clinical relapse = presence of recurrence of symptoms at 4 weeks follow-up, NR - not recorded.

Table S6. Complications/Adverse events of included studies

Study	Complications/Adverse events (N)	Percentage of patients complaining of complications/adverse events out of total sample size
Steube et al., 2017[14]	None	0%
El Nachef et al., 2016[16]	None	0%
Landy et al., 2015[13]	Nausea (3), Abdominal pain (2), Vomiting (1), Fever (1)	87.5%
Herfarth et al., 2019[18]	None	0%
Kousgaard et al., 2020[15]	Nausea (3), Abdominal pain (2), Fatigue (2)	77.8%
Selvig et al., 2020[5]	Abdominal pain (11), Fatigue (3), Nausea (2)	93.8%
Karjalainen et al., 2021[17]	Fever (4), Nausea (3), Abdominal pain (1)	61.5%

Table S7. Table of sensitivity analysis

Excluded Study	Pooled ES	LCI 95%	HCI 95%	Cochran Q	p-value	I2	I2 LCI 95%	I2 HCI 95%
Steube et al., 2017[14]	0.183	0.008	0.359	15.470	0.004	74.144	35.833	89.581
El Nachef et al., 2016[16]	0.066	-0.011	0.143	3.218	0.522	0.000	0.000	74.153
Landy et al., 2015[13]	0.137	-0.022	0.296	16.449	0.002	75.683	40.374	90.082
Herfarth et al., 2019[18]	0.185	0.008	0.361	15.201	0.004	73.686	34.474	89.433
Kousgaard et al., 2020[15]	0.178	-0.014	0.371	16.323	0.003	75.494	39.821	90.021
Selvig et al., 2020[5]	0.153	-0.035	0.341	16.427	0.002	75.650	40.280	90.072

Figure S2. Publication bias assessment. Egger's regression test was used for the 7 included studies.

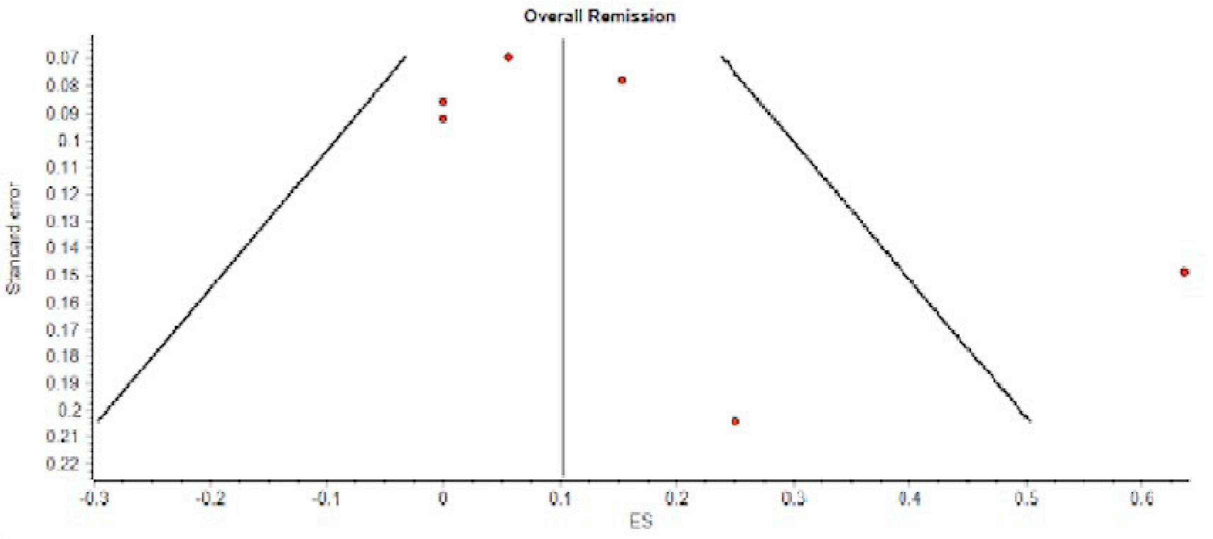

Supplement: Supplementary file 1 [file microorganisms-12-02430-s001.zip › microorganisms-3245445-supplementary.pdf]
